# Exploring masticatory performance: a bibliometric analysis of studies published in the Web of Science database from 1950 to 2024

**DOI:** 10.1590/1807-3107bor-2025.vol39.037

**Published:** 2025-04-04

**Authors:** Luciano José Pereira, Adriana Pinto Bezerra, Aurélio de Oliveira Rocha, Sarah Ferreira Mattos Alcântara, Rayene Cardoso Rodrigues, Mariane Cardoso Carvalho, Thais Marques Simek Vega Gonçalves

**Affiliations:** (a) Universidade Federal de Lavras – UFLA, Department of Medicine, Lavras, MG, Brazil.; (b) Universidade Federal de Santa Catarina – UFSC, Department of Dentistry, Florianópolis, SC, Brazil.

**Keywords:** Mastication, Digestive System and Oral Physiological Phenomena, Bibliometrics

## Abstract

Objectively evaluating an individual’s capacity for food fragmentation (masticatory performance) is crucial for understanding oral physiology and dental rehabilitation processes. Our aim was to conduct a bibliometric analysis of the literature focusing on masticatory performance. We conducted a survey in Web of Science up to August 31, 2024, applying specific category filters. Two calibrated reviewers manually tabulated the data, extracting information on title, authorship, keywords, institutions, countries, number of citations, year of publication, journal title, study design, masticatory test, population, and dentition status. VosViewer software generated collaborative network maps, while JAMOVI was used to perform ANOVA and Poisson regression analyses. The selection process resulted in 814 articles published between 1950 and 2024, with a recent increase to at least 50 articles per year. The average impact factor was 3.16, with around 30 citations per article. Citations were significantly influenced by the masticatory performance technique, publication year, and journal impact factor. The comminution test was the most prevalent (n = 411). Most studies focused on adults (n = 420) and older adults (n = 361), in which natural dentition (n = 404) and complete dentures (n = 214) were the most widely assessed parameters. The most frequent study designs were cross-sectional (n=489) and prospective (n = 145). The leading countries were Japan (n=202), Brazil (n=134), and the Netherlands (n = 69), and the Journal of Oral Rehabilitation was the most recurrent journal (n = 162). This study highlights the growing interest in evaluating masticatory performance, with a significant increase in publications over the years. This study highlights the need for further research involving children and longitudinal study designs, as well as studies evaluating rehabilitations with removable partial denture and implant-supported prostheses.

## Introduction

Mastication represents the initial step of the digestive process and relies on the synchronized actions of various facial muscles.^1^ This coordination results in rhythmic mandibular movements that effectively manipulate and crush food, while saliva moistens food to form a cohesive bolus.^1^ Variations in food consistency and chemistry activate sensory receptors, evoking adequate forces for chewing.^2^ During mastication, aromas, textures, and flavors are perceived within the context of environmental and cultural aspects that contributes to the pleasure of eating.^1,2^

Masticatory function can be analyzed in various groups: e.g., children,^3^ adolescents,^4^ adults,^5^ and older adults.^6-8^ Moreover, investigations can include dentate individuals,^9^ brace wearers,^10,11^ or prosthetically rehabilitated patients;^12,13^ in addition to patients with chronic^14^ or acute^15^ pain conditions; or those taking medications affecting motor control or salivary flow,^16^ among others.

Masticatory function can be assessed using different methods (objectively or subjectively), each one providing distinct parameters that characterize the masticatory process.^17^ Objective methods for masticatory evaluation most frequently include analyzing food comminution capacity or the ability of mixing colored chewing gums or waxes, while subjective methods involve questionnaires and interviews.^18^ Recently, a study involving an international group of experts established a consensus on the terminology used and defined masticatory performance as the individual’s ability to grind or pulverize a specimen of test food after a predetermined number of mastication cycles.^19^ This definition closely aligns with comminution tests using single or multiple sieves, two-color mixing ability tests with chewing gum or wax cubes, and masticatory performance tests with gummy jelly or encapsulated granules.^19^

The comminution tests generally involve the chewing of brittle foods such as nuts and raw carrots or artificial test foods (e.g., silicon-based impression materials such as Optosil/Optocal or irreversible hydrocolloids) and the subsequent determination of the median particle size by passing the resulting fragments through a series of sieves with different mesh sizes.^19,20^ On the other hand, gummy jelly is chewed for a set number of cycles, and the concentration of glucose or ß-carotene in the saliva is measured.^21^ In addition, to determine the mixing ability, volunteers chew a two-colored chewing gum or wax cube for a specific number of cycles. The degree of color mixing is then assessed using visual or digital methods.^22,23^ The encapsulated granule technique quantifies the dye concentration (fuchsine, erythrosine, or adenosine triphosphate) after chewing for a determined number of cycles.^24^ During mastication, the pigment-coated granules encapsulated in the rubber or PVC capsule are ground and a spectrophotometer is used to precisely measure masticatory performance.^24,25^ Masticatory performance key determinants rely on the number of functional tooth units and bite force, underscoring the critical role of preserving these elements in maintaining a healthy functional capacity.^26^

Over the past 75 years, thousands of studies have been conducted objectively evaluating mastication. However, no study has been undertaken to quantify the most frequently employed techniques, the countries and institutions mostly involved in this type of evaluation, the interaction among researchers, the journals that publish most on the topic, and the density of their citations. A bibliometric evaluation of masticatory function is relevant to present objective indicators, tracking the progression of the research over time, highlighting the most prolific authors and institutions, and revealing the most established collaborative networks among researchers.^27^

The objective evaluation of masticatory function provides valuable information on the effectiveness of the chewing process, which is essential for proper digestion and overall well-being.^1^ For dental professionals, objective evaluation allows for the identification of functional impairments that may not be evident through routine clinical examinations.^28^ Consequently, the application of masticatory performance analysis enhances the ability to develop targeted treatment plans, improve patient outcomes, and promote optimal oral health.^29^ Therefore, the objective of this study was to assess the evolution and current status of scientific production on masticatory performance by a bibliometric analysis of articles indexed in the Web of science Core Collection (Clarivate Analytics, Philadelphia, USA).

## Methods

### Search strategy

The search was conducted until August 31, 2024 in the Web of Science Core Collection database using the following strategy: [TS=(“masticatory performance” OR “mastication” OR “masticatory”)]. To narrow the search, category filters such as dentistry, oral surgery, medicine or geriatrics, gerontology or food science, technology or nutrition, dietetics or gerontology, physiology and behavioral sciences were applied. Furthermore, references were filtered by document type to include only articles or review articles.

### Eligibility criteria

Only articles involving the objective evaluation of masticatory performance were included, regardless of the technique used. No restrictions were applied regarding language or year of publication. Articles unrelated to the topic, incomplete records, case reports, in vitro and/or animal studies, conference papers, and editorials were excluded.

### Data selection and extraction process

The articles were first selected by two calibrated researchers (A.P.B. and T.M.S.V.G.) by title and abstract assessment to select only articles in which objective masticatory performance was assessed. When necessary, full-text reading was also performed to clarify any ambiguities. Disagreements were resolved by discussion with a third reviewer (L.J.P.).

The data were tabulated and organized (Microsoft Excel, Microsoft, USA). The following data were extracted from each article: number of citations, year of publication, journal, impact factor (IF) (2023) according to Journal Citation Reports - Clarivate Analytics, study design, masticatory performance test, population and dentition status, country and continent, and institution (based on the corresponding author’s affiliation). To ensure that the publications were attributed to the correct authors, citations with similar names were checked on the institutions’ websites, thereby ensuring accurate grouping of the articles with their respective authors. The same was applied for institutions.

Study designs were categorized as randomized clinical trials, cross-over, prospective, cross-sectional, case-control, or reviews (systematic, scoping, or narrative). Papers were also grouped according to the masticatory performance tests into comminution, mixing ability (chewing gum and wax cubes), gummy jelly, and capsules. Dentition status was also grouped into dentate/partially dentate, complete denture, overdenture, implant-supported complete denture, removable partial denture, and implant-supported removable partial denture.

### Data analysis

The study focused on the production of scientific literature, examining the evolution of scientific productivity over time, authorship, institutions, countries, journals, and techniques used to determine masticatory performance. The 20 most productive authors, along with their respective numbers of publications and individual H indices, as well as the journals and institutions with the highest number of publications in the field, were determined. The numbers of articles per year, per technique, per country, per type of experimental design, and per type of dentition within each technique were also determined.

Bibliometric network maps were constructed using the Visualization of Similarities Viewer software (VOSviewer, version 1.6.17.0, Netherlands) for author density, collaborative co-authorship, and keywords. In the visual outputs produced by the software, terms linked to larger clusters and sources exhibited higher frequencies of occurrence, whereas terms linked to smaller clusters or sources demonstrated lower frequencies of occurrence. The lines connecting the terms represent instances of collaboration or associations between them.

Differences in quantitative characteristics such as mean impact factor, number of citations in Web of Science (WoS), and in all databases among techniques and according to the decade of publication were analyzed using one-way ANOVA followed by Tukey’s post-hoc test. Likewise, influencing factors associated with the number of citations in the WoS database were determined by Poisson regression. A p-value of less than 0.05 was considered to indicate statistical significance (JAMOVI 2.3.28). The normality of the variable distribution was assessed using the Kolmogorov-Smirnov test.

## Results

**Figure 1:**
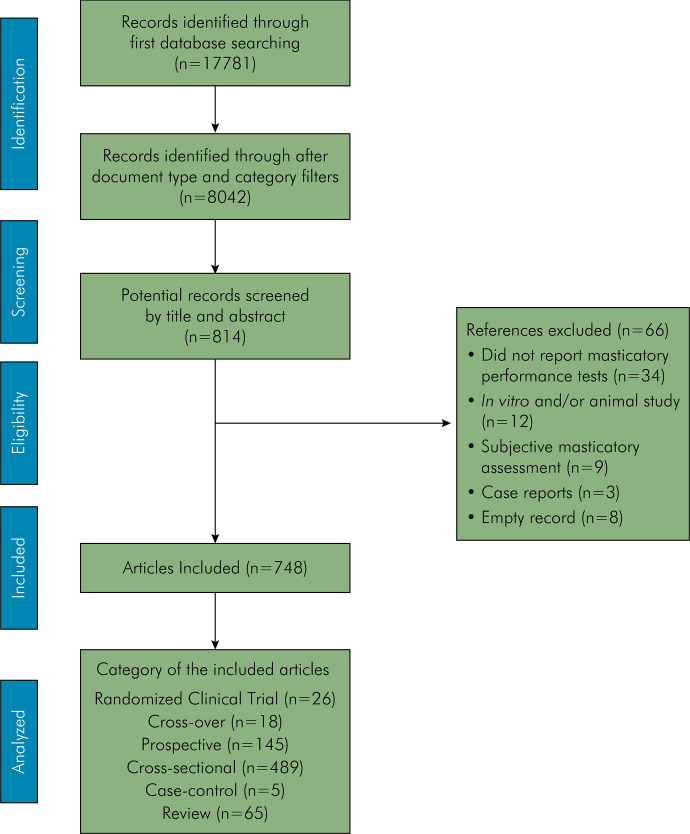
Flowchart showing the study selection protocol.

The primary database search yielded 17,781 articles. After category and document type filter application, only 8,042 references were maintained. After title and abstract screening, some additional articles had to be excluded because they were not reporting data of objective masticatory performance. Thus, only 814 references were exported for data extraction. An additional screening was performed to exclude studies involving animal samples, those that assessed only subjective masticatory parameter, or did not specifically report masticatory performance results. At the end of the study selection process, 748 articles were included. Cross-sectional (n = 489) and prospective (n = 145) designs were the most prevalent, followed by review articles (systematic, scoping, or narrative) (n = 65), randomized clinical trials (n = 26), crossover (n = 18), and case-control (n = 5) studies (Figure 1).

### Masticatory performance techniques

Among the techniques employed for analyzing masticatory performance, comminution tests (n = 411) were the most frequent, followed by mixing ability (n = 268), gummy jelly (n = 133), and capsule techniques (n = 21). Most articles investigated the mastication of adults and older adults in cross-sectional designs (n = 553) (Table 1).

**Table 1:** Distribution of the studies according to the main masticatory tests, study designs, population age, origin of the research, and dentition characteristics.

Test	Study design	Population age			Origin of the research			Dentition characteristics					
		Children / Adolescents	Adults	Older adults	Brazil	Japan	Netherlands	Dentate/ Partially dentate	Complete denture	Overdenture	Implant-supported complete denture	Removable partial denture	Implant-supported removable partial denture
Comminution	RCT	0	6	10	4	0	3	1	8	4	0	2	0
	Cross-over	0	2	7	4	2	0	2	6	3	0	0	0
	Prospective	5	45	55	33	5	7	24	42	16	6	11	5
	Cross-sectional	26	178	72	54	23	30	172	61	14	3	19	0
	Case-control	1	2	2	2	0	0	3	1	0	1	0	0
	Total	32	233	146	97	30	40	202	118	37	10	32	5
Mixing Ability	RCT	0	3	7	2	4	1	2	4	3	0	0	0
	Cross-over	0	2	8	0	2	1	1	5	5	1	1	1
	Prospective	3	10	35	2	13	7	11	13	11	1	6	2
	Cross-sectional	12	94	92	9	59	16	104	42	8	3	23	2
	Case-control	0	0	2	0	1	0	0	1	0	1	0	0
	Total	15	109	144	13	79	25	118	65	27	6	30	5
Gummy Jelly	RCT	0	0	3	0	2	0	0	2	1	0	0	0
	Cross-over	0	1	0	0	1	0	1	0	0	0	0	0
	Prospective	0	7	12	0	10	0	5	4	2	0	2	1
	Cross-sectional	4	56	50	0	87	2	66	20	1	0	12	3
	Case-control	0	0	0	0	0	0	0	0	0	0	0	0
	Total	4	64	65	0	100	2	72	26	4	0	14	4
Capsules	RCT	0	1	2	2	0	0	0	2	1	0	0	0
	Cross-over	0	0	0	0	0	0	0	0	0	0	0	0
	Prospective	0	4	2	1	4	0	4	1	1	0	0	0
	Cross-sectional	1	9	2	8	3	0	8	2	0	0	2	0
	Case-control	0	0	0	0	0	0	0	0	0	0	0	0
	Total	1	14	6	12	7	0	12	5	2	0	2	0

Regarding dentition status, most studies on masticatory performance studies investigated volunteers with natural dentition (including complete and partially dentate individuals) (n = 404). Considering only studies involving prosthetic rehabilitation, complete dentures (n = 214) were most frequently studied, followed by removable partial prostheses (n = 108) and overdentures (n = 70) (Table 1).

### Keywords

**Figure 2:**
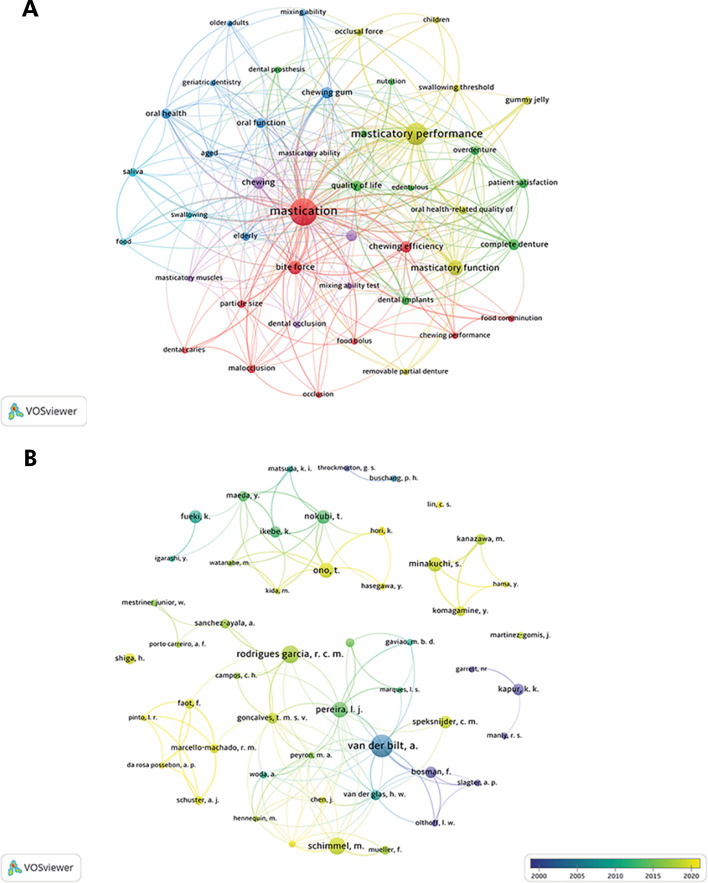
A. Frequency and interaction of the main keywords associated with the studies. Minimum number of keywords: 8 studies. B. Author density map and collaborative co-authorship among them. Minimum number of occurrences for an author: 8 articles.

The criterion of at least eight studies indicated mastication (252 occurrences), masticatory performance (155 occurrences), masticatory function (65 occurrences), and bite force (48 occurrences) as the most preferred keywords (Figure 2A), among others related to dentition (complete denture, malocclusion, dental occlusion, etc.).

### Countries and continents

**Figure 3:**
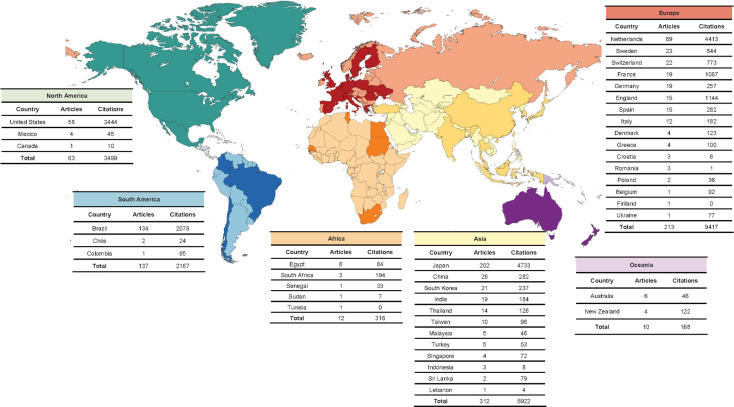
Worldwide distribution of the origin of publications on masticatory performance and the respective number of citations.

Most studies were primarily conducted in Japan (n = 202), Brazil (n = 134), the Netherlands (n = 69), the United States (n = 58), Sweden (n = 23), and Switzerland (n = 22). The studies performed in Brazil and in the Netherlands focused more on comminution tests, while Japan, Sweden, and Switzerland published more studies on mixing ability and gummy jelly techniques (Figure 3, Table 2). In general, publications on masticatory performance have been carried out by groups from all continents, with most of them published by groups in Asia (n = 312) and Europe (n = 213), followed by South America (n = 137) and North America (n = 63) (Figure 3).

**Table 2:** Authors and journals with more publications on masticatory performance.

Top 20 Authors					Top 20 Journals		
Author	Institution, Country	Number of articles	Number of citations WoS	H-Index	Journal	Number of articles	Impact factor
Bilt, A.	University Medical Center Utrecht, Netherlands	42	3676	47	Journal of Oral Rehabilitation	162	3.1
Rodrigues Garcia, R.C.M.	Campinas State University, Brazil	30	438	26	Journal of Prosthetic Dentistry	60	4.3
Schimmel, M.	University of Bern, Switzerland	29	888	37	Journal of Prosthodontic Reseach	41	3.2
Pereira, L.J.	Federal University of Lavras, Brazil	26	836	30	Archives of Oral Biology	38	2.2
Ono, T.	Osaka University, Japan	25	440	26	International Journal of Prosthodontics	35	2.1
Minakuchi, S.	Tokyo Medical and Dental University, Japan	23	469	33	Journal of Dentistry	26	4.8
Nokubi, T.	Osaka University, Japan	21	920	26	Gerodontology	21	2.0
Fueki, K.	Tokyo Medical and Dental University, Japan	20	639	23	Clinical Oral Implants Research	20	4.8
Speksnijder, C.M.	University Medical Center Utrecht, Netherlands	19	361	20	Journal of Dental Research	16	5.7
Ikebe, K.	Osaka University, Japan	17	793	35	Physiology & Behavior	13	2.4
Bosman, F.	University Medical Center Utrecht, Netherlands	17	1532	41	Journal of Prosthodontics	13	2.1
Kapur, K. K.	University of California Los Angeles, United States of America	17	793	28	Journal of Texture Studies	12	2.8
Kanazawa, M.	Tokyo Medical and Dental University, Japan	16	421	24	Clinical Oral Investigations	12	3.1
Shiga, H.	Nippon Dental University Tokyo, Japan	16	267	13	Acta Odontologica Scandinavica	11	1.4
Gonçalves, T.M.S.V.	Federal University of Santa Catarina, Brazil	16	261	15	Journal of Oral Science	9	1.9
Maeda, Y.	Osaka University, Japan	15	802	42	International Journal of Oral and Maxillofacial Implants	10	1.7
Glas, H.W.	Zhejiang Gongshang University, China	15	849	30	Cranio-The Journal of Craniomandibular & Sleep Practice	8	1.6
Komagamine, Y.	Tokyo Medical and Dental University, Japan	14	274	14	Nutrients	7	4.8
Faot, F.	Federal University of Pelotas, Brazil	14	169	21	American Journal of Orthodontics and Dentofacial Orthopedics	6	6.5
Marcello-Machado, R.M.	Federal University of Pelotas, Brazil	13	147	13	Angle Orthodontist	6	3.0

### Authors and institutions

A total of 2,360 authors contributed to articles on masticatory performance. Table 2 presents the top 20 authors based on number of publications and their respective institutions; van der Bilt, A (n = 42) was the author with the highest number of publications, followed by Rodrigues Garcia, R.C.M. (n = 30), Schimmel, M. (n = 29), Pereira, L.J. (n = 26), and Ono, T. (n = 25). The five authors with more publications are from four different continents: Europe (the Netherlands in first place and Switzerland in third place), South America (Brazil in second and fourth places), and Asia (Japan in fifth place). Almost all the other authors in the top 20 list belong to the same countries and/or share collaborations (Figure 2B, Table 2). Consequently, the institutions with the highest number of publications also belong to the same countries: Japan, the Netherlands, Brazil, and Switzerland (Table 2).

### Year of publication

**Figure 4:**
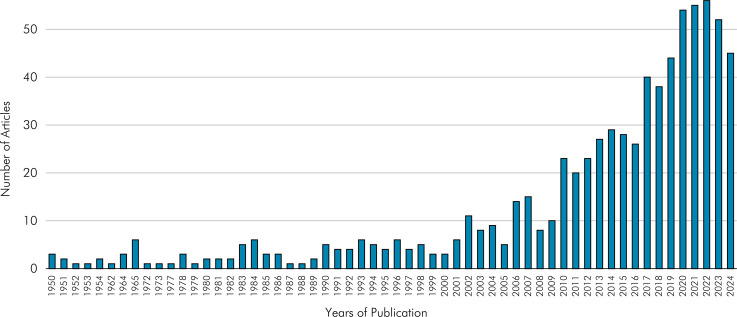
Distribution of the number of publications per year.

The oldest article was published in May, 1950, titled “The effect of dental deficiency on mastication and food preference” by Manly, R.S and Shiere, F.R., published in Oral Surgery Oral Medicine Oral Pathology Oral Radiology and Endodontics.^30^ The distribution of the number of articles published over the years (since 1950) has impressively increased, going from three articles in 1950 to more than 50 per year after 2020 (Figure 4). The 2011-2020 decade had the highest number of publications (n = 343), more than twice the number of the previous decade. For the current decade, the expectation is for another increase, considering that we are still in the fourth year of the decade and there are already more than 200 publications on masticatory performance (Table 3).

**Table 3:** Number of publications, average impact factor, and citations in the Web of Science and all databases according to each decade, starting from 1950.

Decade of publication (number of articles)	Impact factor mean ± SD (min.–max.)	Number of citations – WoS mean ± SD (min.–max.)
2021–2024 (n = 204)	3.11 ± 1.47 (0–10.2)^c^	3.67 ± 5.82 (0–62)^d^
2011–2020 (n = 343)	3.17 ± 1.15 (0–10.7)^c^	23.2 ± 27.9 (1–320)^cd^
2001–2010 (n = 116)	3.40 ± 1.80 (0–10.7)^bc^	61.4 ± 73.4 (3–564)^b^
1991–2000 (n = 46)	3.90 ± 2.15 (0–8.9)^b^	74.0 ± 79.6 (3–523)^ab^
1981–1990 (n = 34)	3.29 ± 1.34 (0–6.6)^bc^	54.8 ± 52.9 (0–210)^b^
1971–1980 (n = 9)	3.61 ± 1.86 (0–6.3)^b^	44.0 ± 49.4 (5–147)^bcd^
1961–1970 (n = 10)	4.60 ± 0.0 (4.6–4.6)^ab^	49.3 ± 41.3 (14–115)^b^
1951–1960 (n = 6)	6.57 ± 2.59 (3.5–8.9)^a^	34.2 ± 21.6 (7–72)^bcd^
1941–1950 (n = 3)	3.97 ± 4.50 (0.1–8.9)^abc^	144 ± 146 (44–311)^a^

One-way ANOVA and Tukey’s tests. Different lowercase superscript letters in the same column denote statistical difference (p <0.05). ^*^Articles in which more than one technique was employed were duplicated to account for the number of times each technique was used.

### Journals and impact factor

The Journal of Oral Rehabilitation (n = 162) was the journal that published the highest number of articles related to masticatory performance, followed by the Journal of Prosthetic Dentistry (n = 60), and the Journal of Prosthodontic Research (n = 41). The 20 journals that most published articles on masticatory performance were those whose primary scope involves dentistry, dental prosthetic rehabilitation, oral physiology, and gerontology, with impact factors ranging from 1.4 to 6.5 (Table 2). The average impact factor of publications in this area was 3.16, with approximately 30 citations in WoS. The mean impact factor among all masticatory performance techniques was similar (p > 0.05), (Table 4).

**Table 4:** Comparison of impact factor values, number of citations in Web of Science and all databases, considering different techniques* for determining masticatory performance.

Masticatory test (number of studies)	Impact Factor - Journal Citation Report mean ± SD (min.–max.)	Number of citations in Web of Science database mean ± SD (min.–max.)
Comminution (n = 349)	3.16 ± 1.41 (0–11)^a^	35.3 ± 52.2 (0–523) ^a^
Mixing ability (n = 220)	3.04 ± 1.09 (0–6.6)^a^	19.2 ± 25.2 (0–157)^b^
Gummy jelly (n = 118)	2.80 ± 1.07 (0–6.3)^a^	20.0 ± 26.4 (0–128)^b^
Capsules (n = 19)	2.75 ± 0.87 (1.5–4.8)^a^	30.5 ± 23.0 (2–73)^ab^
Review (n = 64)	3.24 ± 1.89 (0–11)^a^	48.7 ± 91.2 (0–564)^a^

One-way ANOVA and Tukey’s tests. Different lowercase superscript letters in the same column denote statistical difference (p < 0.05). ^*^Articles in which more than one technique was employed were duplicated to account for the number of times each technique was used.

### Citation analysis

The articles received a total of 21,646 citations in the WoS-CC. The most cited article, with 564 citations, was “Food oral processing - A review” by Chen, J.S.;^31^ followed by “Biting and chewing in overdentures, full dentures, and natural dentitions” by Fontijn-Tekamp, F. A., et al.,^32^ with 523 citations and “Determinants of masticatory performance in dentate adults” by Hatch, J.P. et al.,^26^ with 320 citations. The number of citations for the evaluated articles was significantly influenced by the technique used to determine masticatory performance, and it was lower for articles that employed gummy jelly and mixing ability in comparison to the comminution test (p < 0.001), as well as the year of publication in which the older the article (i.e., the earlier the publication year), the higher the number of citations (p < 0.001). The impact factor showed a positive relationship with the number of citations for articles involving masticatory performance, in which the higher the impact factor (p<.001), the higher the number of citations (Table 5).

**Table 5:** Influencing factors associated with the number of citations in the Web of Science database (Poisson regression model).

Variables				95% Exp(B) Confidence Interval		Significance Metrics	
Effect	Estimate	SE	exp(B)	Lower	Upper	z	p-value
Intercept	32.018	0.01250	24.577	23.978	25.182	256.235	< 0.001
Masticatory test							
Mixing ability - Comminution	-0.2402	0.01914	0.786	0.757	0.816	-12.546	< 0.001
Gummy jelly - Comminution	-0.2162	0.02340	0.806	0.769	0.843	-9.240	< 0.001
Capsules - Comminution	0.0394	0.04274	1.040	0.956	1.130	0.922	0.357
Impact Factor	0.0839	0.00537	1.088	1.076	1.099	15.618	< 0.001
Year of Publication	-0.0258	3.89e-4	0.975	0.974	0.975	-66.267	< 0.001

SE: Standard error.

## Discussion

Bibliometric reviews are important tools to provide a comprehensive overview of a research field, identifying gaps in knowledge and suggesting new research directions.^27^ These reviews direct future studies into more targeted and relevant subjects within an area of research.^27,33^ Ultimately, they lead to advancements in clinical practice and improved patient outcomes by highlighting deficiencies in existing research and paving the way for innovative solutions and therapies.^33^ The findings of this bibliometric analysis highlight the global distribution of research and underscore the importance of evaluating masticatory function.

Regarding the most prevalent keywords, the finding was expected, given that masticatory performance is strongly related to the masticatory process itself.^17,26^ Additionally, the masticatory process is mainly influenced by dentition, jaw muscle activity, tongue function, lip strength, and bite force.^28^ Comminution tests are closely related to both maximum voluntary bite force and dental condition,^26^ and considering that this was the most prevalent technique among the retrieved studies, these keywords were expected to appear more often. Bite force is one of the most important determinants of masticatory function and, alone, it accounts approximately for 40% of the variation in masticatory performance.^9,26^

The number of articles using objective methods to evaluate mastication, specifically employing masticatory performance techniques, has been quite substantial, reaching approximately 750 clinical studies, along with an additional 10% of literature review articles. The cross-sectional modality was the most prevalent, and this was also expected because observational studies are widely conducted in health care research.^34^ In this type of scientific report, investigators do not interfere with the phenomena under study. Instead, they observe the phenomenon in a systematic and standardized manner, collecting and recording information, data, or materials that occur at a particular point in the health-disease process or throughout its natural evolution.^34^ The present study focused specifically on the objective evaluation of masticatory function, without incorporating subjective assessments (self-reported perceptions), as these two types of analysis often show limited concordance.^35^ Generally, patients’ perceptions tend to be more optimistic than their actual ability to fragment food.^28^ Thus, a dedicated bibliometric analysis on this topic may be more suitable.

Considering the number of published studies employing each technique, the highest number was observed for comminution, followed by mixing ability and gummy jelly, with very close numbers, while capsules presented the lowest number of articles in comparison to the other techniques. We believe this number was potentially influenced by the time of creation of each method, as the earliest articles on comminution were published in 1950,^18,30,36^ while the other techniques appeared only at the beginning of the 1980s,^37,38^ that is, more than 30 years later.

The number of studies conducted by each country or continent is related initially to the universities where the techniques were developed. The first studies on comminution found in WoS Core Collection originated from the Tufts University School of Dental Medicine, located in Boston, Massachusetts, USA.^18,30,36^ Until the 1970s, nearly all published studies came from this university, with rare exceptions, mainly from Japan^39^ and the Netherlands.^37^ On the other hand, the first study citing the use of the technique known as mixing ability dates back to 1982, conducted in London,^40^ whereas the gummy jelly technique was first reported by authors from Sweden,^41^ and the capsule technique by authors from Japan.^38^

The comminution technique has gained widespread use, with notable contributions from the Dutch group at Utrecht Medical Center,^20^ which established various prolific partnerships, especially with Brazilian researchers.^1,17^ The mixing ability technique also disseminated, notably through a Swiss group, which frequently collaborated also with Brazilian researchers.^12,42^ The gummy jelly technique has been predominantly used in Japan, especially at Osaka University, with some partnerships with the United States.^21,43^ Similarly, the capsule technique is associated with Japanese and Brazilian research groups.^24,25,44^ However, it is worth noting that all techniques are widespread across various groups, including studies that employ combined techniques.^45^

Most articles investigated mastication in young and older adults, followed by studies on older people, likely due to the significant relevance of chewing assessments in relation to tooth loss and rehabilitations, which are more common in these age groups.^46^ The average number of missing teeth increases with age.^47^ Fortunately, there has been a documented decline in edentulism along the past decades.^48^ For younger patients, studies typically focus on malocclusion or orthodontic therapy, as well as on painful conditions of the temporomandibular joint (TMJ) and facial muscles.^3,49^

The average impact factor of publications involving clinical data collection on masticatory performance was approximately 3, with no differences among the studied techniques. This demonstrates that all methods are valid and used in research worldwide.^19^ In comparison to the average impact factor of journals in the fields of dentistry, oral surgery, and medicine,^50^ which was 2.01 with a median of 1.7 (based on the 157 journals listed in the 2023 edition of the Journal Citation Reports), it can be inferred that the field includes significant journals with an impact factor that is 50% higher than the average. The impact factor is calculated by dividing the total number of citations that a journal receives by the total number of articles published within two years (or a five-year period). Consequently, a well-cited journal is more likely to achieve a higher impact factor. Therefore, only journals with widespread access are likely to be highly cited.^51^ The number of citations varied slightly between the techniques, with review studies and those using the comminution technique being slightly more cited. These findings were expected, considering that review articles provide widely used concepts and definitions that serve as theoretical references, while comminution studies have been conducted for a longer period, thus having a higher number of articles employing the technique. This does not include the 20 years during which the technique was almost exclusively used, as mentioned previously.

This study acknowledges certain limitations, including potential biases in citation analysis and the exclusive use of the WoS database within the categories, which might omit papers from other sources. However, the WoS database is widely regarded as one of the most critical sources for bibliometric analysis, offering comprehensive and reliable access to scholarly research across various disciplines. This facilitates high-quality academic studies and informed decision-making. Additionally, we attempted to utilize various categories (dentistry, oral surgery, medicine, geriatrics, gerontology, food science, technology, nutrition, dietetics, physiology, and behavioral Sciences) to encompass all areas that directly or indirectly conduct research on masticatory performance, thereby minimizing the risk of selection bias. Self-citations and citations from books or non-English journals were not considered. Despite these limitations, the data offer significant insights into objective methods for masticatory evaluation trends over almost 75 years.

From the present study, we were able to highlight some important aspects for research groups around the world working on the assessment of masticatory function, in order to guide new studies, which are still scarce. For example, more than 90% of existing studies focused on adults or older individuals, with a much smaller portion conducted on children. Additionally, over 70% of the selected studies had a cross-sectional design, highlighting the scarcity of longitudinal studies. The techniques of gummy jelly and capsules are still less commonly used globally, and they are more restricted to Asian groups. Most studies focus on natural dentition or total prosthetic rehabilitations, indicating the need for new studies on removable partial rehabilitations and implant-supported prostheses.

In conclusion, this bibliometric analysis highlights a growing interest in the objective evaluation of masticatory performance, with a significant increase in publications over the years. The findings underscore the global distribution of research and emphasize the importance of evaluating masticatory function. This study underscores several key points for research groups globally engaged in the evaluation of masticatory function, providing valuable direction for future studies, which remain limited in scope.
